# Knowledge and Health Seeking Behaviour of Breast Cancer Patients in Ghana

**DOI:** 10.1155/2019/5239840

**Published:** 2019-04-01

**Authors:** Francis Agbokey, Elorm Kudzawu, Mawuli Dzodzomenyo, Kenneth Ayuurebobi Ae-Ngibise, Seth Owusu-Agyei, Kwaku Poku Asante

**Affiliations:** ^1^Kintampo Health Research Centre (KHRC), Ghana Health Service, P.O. Box 200, Kintampo-BA, Ghana; ^2^School of Public Health, College of Health Sciences, University of Ghana, Accra, Ghana; ^3^Korle-Bu Teaching Hospital, Accra, Ghana; ^4^Institute of Health Research, University of Health and Allied Sciences, Ho, Ghana

## Abstract

**Background:**

Breast cancer is a major contributor to cancer-related deaths among women worldwide, despite the numerous measures employed to prevent and manage the disease. This study explored the knowledge and health seeking behaviour of breast cancer patients at the Komfo Anokye Teaching Hospital.

**Methods:**

A descriptive cross-sectional study was conducted at Komfo Anokye Teaching Hospital in Kumasi, Ghana, from June 2014 to July 2014. Thirty-five participants were purposively selected. The responses to questions about their experiences with breast cancer were determined using indepth interviews. Transcripts were coded and analysed using NVIVO version 10.0.

**Results:**

Participants' knowledge about signs and symptoms of breast cancer after their diagnosis was high but low for risk factors. Screening for breast cancer through self-breast examination was infrequently performed prior to their diagnosis. The patients' first point of care was generally health facilities. Some patients reported late due to misinterpretation of signs and symptoms, cultural influences and fear of losing their breast to surgery, physician delay, health providers' laxity, and disinterest in breast cancer. Men, for example, husbands, decide on where and when breast cancer patients go for treatment.

**Conclusion:**

There is poor knowledge of the risk factors for developing breast cancer. Patients resorted to the hospital as first options for cure but were generally delayed in doing so. There is the need to create awareness about breast cancer among the general population.

## 1. Introduction 

Throughout the world, breast cancer (BC) has been identified as a major public health problem and the leading cause of cancer-related deaths among women, particularly in low-resourced regions of the world [1, 2]. Globally, about 2.1 million women were estimated to have BC in 2018 and, among these, estimated 626,679 BC-related deaths were reported. This represented an increase of about 19% and 17% in the incidence and mortality rates, respectively, between 2012 and 2018 [1, 3–5]. Ghana has no national cancer register. However, reports by World Health Organisation (WHO) indicate that breast cancer is the second commonest cancer type, constituting 16% of cancers plaguing women in Ghana [6].

Whereas developed countries have much higher incidence rates than their counterparts in less developed and poor-resourced regions across the globe, the corresponding mortality rates are low. For instance, while Africa recorded 168,690 cases of diagnosed BC in 2018 with 74,072 (44%) deaths occurring, North America is reported to have recorded 262,347 diagnosed cases of BC and 46,963 (18%) deaths [1]. This lower-incidence but higher-mortality is attributable to an amalgamation of factors including late detection, inadequate access to appropriate treatment services, and cultural barriers which result in under reporting of BC cases in Africa [7–10].

The term “health-seeking behaviour (HSB)" for purposes of this study is defined as a series of curative activities that individuals embark on as they seek cure of their perceived ill-health. This act is initiated by problems that can be defined as symptoms which in turn lead to the planning of treatment [11]. Reports revealed that majority of BC patients use other forms of treatment than the biomedical treatment of BC and which may pose serious problems to further biomedical management in the long term [12, 13].

Knowledge of breast cancer goes a long way in determining HSB [14]. The term “patient delay (PD)” as used in this study refers to the period from recognition of the first symptom by the patient to initial medical consultation, usually spanning a period of more than three months [15, 16]. Since information is essential for making informed decision, women with BC are said to have substantial needs for relevant and appropriate information throughout their BC journey.

The purpose of this study was to explore the health seeking behaviour of BC patients and their knowledge of BC in a breast cancer management Centre of Komfo Anokye Teaching Hospital in Ghana.

## 2. Methods

### 2.1. Study Design

The study design was a qualitative exploratory descriptive one. This approach was adopted in order to get a deeper understanding of the participants' health seeking behaviours and experiences of breast cancer.

### 2.2. Study Location

The study was conducted at KATH, located in Kumasi, the second largest city of Ghana [17, 18]. KATH was chosen for this study because it is a large referral hospital for the Ashanti Region as well as the second largest teaching hospital in Ghana. With a 1000 bed-capacity, the hospital provides care for a catchment population of approximately 14 million in the middle and northern belts of Ghana. KATH runs a daily breast cancer clinic [19]. BC constitutes 24% of all cancer cases diagnosed and managed by KATH [19]. A total of 1,469 cases of BC were diagnosed and managed at the KATH between 2009 and June 2014; the tail end coinciding with the time data was collected for this study. The services rendered at KATH to BC patients include palliative care, chemotherapy, and radiology.

### 2.3. Study Population and Sampling Procedures

The study was conducted among BC patients and their caregivers attending the Out-Patient Department (OPD) of the Oncology and Radiotherapy of the KATH, clinical specialists, and other health workers at the Oncology Directorate of KATH and also herbalists. All patients were purposively selected to participate in the study based on their scheduled visit to the hospital for review or checkup. With the assistance of a clinician's attendant at the records section BC patients who reported for checkup on the days of the survey were approached to seek consent. Those who willingly agreed to participate in the study were then taken to a room made available to the team by the unit and interviewed after they had consented. This procedure was repeated over a ten-day period till the point of saturation was reached with the twentieth (20th) interview. Additionally, eight (8) caregivers of BC patient participants who were present with patients at the time of the study, five (5) health workers comprising three (3) clinical specialists, and two (2) breast care nurses from the oncology unit of KATH were purposively selected and interviewed as caregivers and health care providers, respectively, after they willingly agreed to participate in the study and had consented. Two herbalists who were mentioned as providers of alternative treatment for BC patients were also interviewed after they willingly agreed to participate in the study and had consented.

### 2.4. Data Collection Procedure

Data were collected through indepth interviews (IDIs), to explore issues that relate to health- seeking behaviours among BC patients with focus on knowledge and health seeking behaviour for BC. IDIs were also conducted for selected key informants. A total of 35 IDIs were conducted among all respondents involved in the study between June 14th and July 24th, 2014 as illustrated in Table 1. All interviews were tape-recorded [20].

### 2.5. Data Management and Analysis

The tape-recorded interviews were transcribed verbatim and field notes typed, expanded, and saved as word documents on the day of the interview or shortly thereafter. The data were reviewed each week to identify potential emerging issues from the study. These were then compiled, tabulated, and analysed into themes using NVIVO version 10. The results were finally presented on the overall themes as narratives and supported with quotes.

All interviews were conducted by a Research Officer with a minimum qualification of Bachelors' degree who has also been trained on both the theoretical foundations and the application of relevant qualitative techniques. The tools were developed in English and translated into “Twi” the local language using a back-translation process as quality assurance for participants who were not fluent in English. The tools were pretested at the Peace and Love Hospital located at Oduom, Kumasi in Ashanti Region. The pretesting proved very useful as it afforded the research team the opportunity to fine-tune the interview guide to elicit the appropriate responses from participants.

### 2.6. Ethical Consideration

Ethical approval was obtained from the Ghana Health Service Ethical Review Committee (GHS-ERC); study approval number:* GHS-ERC64/04/14*. Permission was also sought from the Committee on Human Research, Publications and Ethics, Kwame Nkrumah University of Science and Technology, School of Medical Sciences and Komfo Anokye Teaching Hospital in Kumasi, Ghana, as well as from the Oncology Directorate of KATH. Written informed consent was sought from all study participants. Anonymity and confidentiality of study participants was properly adhered to. Interviews were tape-recorded with prior permission of the participants. All participant data and study documents have been kept in locked cabinets.

## 3. Results

From Table 2, the BC patients that participated in this study had ages ranging from 29 to 80 years with a median age of 52.5 years. Seven (85%) patients were between age category of 31 and 60 years. Twelve (60%) patients had completed Basic Education Certificate Examination (BECE). Nineteen (95%) of them reported being of Christian faith; 18 (90%) were married with 11 (55%) of them being engaged in petty trading. All 5 health professionals had tertiary education and the 2 herbalists had basic education as the highest level of education attained.

### 3.1. Experiences with BC Burden

The patients generally perceived BC to be a very dangerous, terrible, and fatal disease which spreads very fast and kills instantly. They believed anyone who chooses to ignore the devastating effects of BC did so at their own peril. This view is illustrated by a patient's response below.

“This BC disease is a killer disease! This is because when it affects you and you do not send it to the hospital, it can lead to your breast being cut off and when that is done, some people may lose their lives through that. Not many people survive from BC.”  (IDI with BCP No. 12)

### 3.2. Perceptions of Causes of BC

Patients held varied perceptions about the causes of BC. Some of them perceived that BC is a punishment meted out by their ancestors or their gods for not having children to save the human race from extinction. They however added that this view seems to have changed lately because BC is common among even women who have given birth to children. This perception is illustrated by the response below.

“I can't tell. I can't tell what it really is, because at first they said it's for people who haven't given birth before as a punishment from the gods and ancestors for their refusal to give birth but as it stands now even those of us with children also have the disease…I really can't tell.”  (IDI with BCP No. 13)

There was one patient who attributed the cause of her BC condition to a spider-bite that led to itching around the nipple and nipple discharge. This view is amply captured by her narrative:

“As for me I don't know. No one in my family has ever gotten it. If that insect had not bitten me I would not have even known about it. It was a spider; very small spider. It came to hide in my dress and when I hit it through the dress it bit me resulting in itching around my nipple and nipple discharge”  (IDI with BCP No. 07)

BC patients generally cited hereditary and lifestyles such as eating of fatty foods as some predisposing factors for the BC disease. Their view is captured by the following quotes below.

“It's about your health and your diet. The things we make our food with sometimes come with a lot of sickness. So watch your diet. Cut down on oily food, and you will be less susceptible to BC. It can also be inherited...”  (IDI with BCP No. 18)

Caregivers of BC patients and herbalists described BC as an abnormal growth in or on the breast. One herbalist likened the growth of BC to that of uterine fibroid, which was said formed slowly and then gradually occupies the entire region of the breast. This view by the herbalists is illustrated with the quotes.

“BC is something which before, was not present in the breast but has now found its way to be present in the breast now. It is just like fibroid; it forms slowly and then occupies the entire region of the breast. As it grows, it is referred to as cancer”  (IDI with herbalist 01).

The herbalists attributed BC to three main practices by some women. These were smearing of oils and other substances on the breast by women in their bid to making the breast look more attractive and keeping of money in the brassieres and the emission of radiation from mobile phones kept close to their breast in their brassieres. This opinion is illustrated by the response below.

“First of all, there are some women who are fond of applying oil or other substances to their breast just so they can make it look more attractive but what they do not know is that, when you apply the oil to the breast and expose it, ... a lot of dust and other unwanted substances can penetrate into the breast, making it very likely for you to get the disease. Also, some women prefer keeping their phones in their brassieres and whenever the phone vibrates, it causes harm to the breast and this is one factor that generates BC rapidly. So in all, the oil women apply to the breast, the monies kept by the breast as well as keeping phones on the breast are all pathways to or causes of getting BC.” (IDI with Herbalist 01)

### 3.3. Participants' Experiences of Signs and Symptoms of BC

The most common signs and symptoms of BC that were mentioned and described by the BC patients include lump in the breast which may either be painful or painless, sharp pain in the breast, itches of the breast, and discharges from the breast and darkening of the face and palms. This view is illustrated by the responses below:

“Your nipples begin to ache, bleeding from the nipple and swelling of the breast, heaviness, hardening and reddening of the breast coupled with itches and sharp pains in the breast”  (IDI with BCP No. 12)

### 3.4. Participants' Perceptions about Treatment and Cure of BC

Some (7) of the BC patients were of the view that the disease was not curable, and there were others who were not so sure and hence were unable to tell with certainty whether it could be cured. There were also those who opined that the BC disease is curable on condition that it was detected early enough. Whereas others believed it could be cured, they could not tell if that cure would be permanent or whether there was the possibility of the disease recurring at some point in the future. These varied responses by the patients are illustrated below.

“Yes, BC can be cured but only when it is detected early. I was told if you seek early treatment, you can be cured. I think when the lump found in the breast is not as big as to cause severe damage to the breast, one can be cured.”  (IDI with BCP No. 14)

There was however a respondent who was of the view that the disease could be managed if physician's instructions about medication are religiously adhered to by the patient; she was however not sure if BC could be completely cured. This view is illustrated by the response below.

“I know that if you follow all the instructions the doctors give you the cancer's effects will reduce. But whether it can go away completely I don't think so.”  (IDI with BCP No. 18)

### 3.5. Sources of Seeking Healthcare for BC

Most (12) of BC patients first sought health care from the unorthodox sources such as traditional healers or herbalists, drug stores, and prayer camps. They also relied on home-made remedies and concoctions. This first phase of health seeking could last for months and is influenced by friends and relatives, for example, husbands and children. They eventually sought care from health centres, clinics, and hospital.

“… I didn't know it was BC, so I first went to a prayer camp upon the advice of my sister-in-law who said she suspected the lumps in my breast were caused by witches and will therefore require spiritual means to combat them. I was only made to fast and pray from 6 am to 6 pm after which I was given anointing oil blessed by the pastor to be smearing on the breast to ward off the witches who have been using my breast as football in the night thereby causing the lumps in my breast. But after three months of going there and not seeing any improvement, my husband advised that I went to the hospital and I did.”  (IDI with BCP No. 06)

There were a couple of patients whose health seeking journey however commenced first in a hospital through unorthodox care by herbalists, eventually ending up at the hospital again. For example, a patient shared her experience of how she had to first seek care at the KATH but later had to stop and go for herbal care for six (6) months due to financial difficulties in meeting treatment cost at KATH. She had however returned to KATH after her children were able to raise some money to pay the medical bills. Her experience is captured below:

“I used to come for treatment here but I stopped for a while because I could not afford the high cost of medication so I was advised by my children to go to seek herbal treatment elsewhere at the Dr. “XX” Herbal Centre and then it deteriorated after almost six months of going there so I stopped and came back here [KATH] after my children had promised to help foot the cost of medication.”  (IDI with BCP No. 07)

### 3.6. Description of the Nature and Type of Care Received at the Hospital

Some of the patients, who sought treatment for their BC at the hospital, described their experiences in relation to the type and nature of care received. A patient who resorted to KATH for care outlined how she had to endure great pain to undergo two separate operations to have lumps in her breast and armpit removed and was now waiting to undergo chemotherapy and radiation as captured below:

“When they saw it and I brought it to my doctor, he also educated me on that and said it's the initial stage. He made me go through all the necessary tests to see whether it has affected any other parts of my organs or body. He saw that I had a lump and that I had another lump under my armpit. So he brought me to the Tumour Board and they asked him to do the surgery for me to remove the lumps. He educated me and made me aware that I may have to go through three different steps namely: surgery, radiation and chemotherapy. After the operation/surgery I need to go through either radiation or chemotherapy. So right now, the first step has been done. I went through the operation twice. The first one that they removed was the lump in my breast, and then upon the second surgery they removed the one under my armpit. As you see me here today, I've come to meet with the Tumour Board again for them to assess the area to enable me start with the radiation therapy probably this coming Friday. But to be honest with you, I'm very scared about the whole process because I've been reliably informed by one of my friends who had gone through this whole thing before that it's very painful. I only pray and hope that I'll have the courage and strength to endure the pain and go through it successfully”  (IDI with BCP No. 01)

Another patient was describing the nature of care obtained at KATH and how she had one of her breasts cut off as well as how some parts of her body such as arms, legs, and face became dark following what she described as a very painful injection she was given as part of the treatment. This is illustrated in the quote below:

“They treated me for about seven months, after which they cut my breast off. ... I have been injected. It's very painful too; my arms, legs and face became very dark or black. I also threw up a lot after undergoing this process.”  (IDI with BCP No. 20)

Some BC patients first went to a private hospital and other clinics at the primary health care level where the nature of care obtained was different from what pertained at KATH which is a well-resourced referral hospital. The experience of a patient is illustrated below:

“I first went to a private clinic to complain to them after noticing a lump in my breast and started experiencing slight pains in that breast. So they had to do surgery to remove it. It started as a lump. One thing that surprised me about the treatment they gave me over there was that, there was no X-ray, no lab test, just straight forward surgery. If they had done X-rays and lab tests, they would have known exactly what was wrong with me. Instead they referred me to KATH to come and get tested after the surgery to remove the lump.”  (IDI with BCP No. 05)

### 3.7. Practice of Self-Breast Examination among Patients

Another reason for the delayed presentation for treatment for breast cancer at the hospital was the infrequent screening for breast cancer which would have ensured that the condition was detected early enough for treatment. Patients who participated in this study reported not to be practicing in such screening methods as breast self-examination (BSE) and clinical breast examination (CBE) and mammogram which are all ways of detecting breast cancer. This view is illustrated by the following quote.

“They [the nurses] checked the breast after which they thought us how to check it ourselves with the hands and they said we should always be checking for lump. Though I do it, I do not do that often.”  (IDI with BCP No. 16)

Health workers were generally of the view that uptake of screening activities such as breast self-examination (SBE), clinical breast examination (CBE), and mammogram to enable early detection and diagnosis of the breast cancer disease was irregular and poor among women despite the advice given to them during health talks at the various departments of the hospital and in the community by health workers to encourage them to do so. This is illustrated by the quote below.

“The delay usually is related to the patients. The patients they don't usually see the cancer at early stage or they don't self-examine with respect to breast cancer, they don't self-examine to detect the cancer at early stage.”  (IDI with H W No. 01)

### 3.8. Factors That Influenced the Choice of Health Care for BC

The decision of choosing which source of healthcare among the array of others was influenced by factors such as the source of information about treatment of BC, influence by significant others or gate-keepers such as spouse, and one's belief and perception about the etiology and causation of the disease condition.

The role of husbands and in-laws in the decision-making process, such as determining where and when to seek health care for BC, was evident as most of the patients who participated in this study mentioned their husbands as the final decision-makers in seeking healthcare for their BC condition. The response below illustrates this.

“…it was my husband's to go and seek health care from the hospital. I did not challenge him on that because he was going to pay the bill.”  (IDI with BCP No. 16)

Another patient (IDI with BCP No. 16) recounting her choice was influenced and shaped by her in-law had this to say: please refer to quote under sources of seeking healthcare for BC.

### 3.9. Duration between Onset of BC and Health Seeking

The duration between the first symptom of BC and first time of seeking care among the BC patients ranged from 3 days to 15 years with most of them seeking care after three months of symptom recognition. This is illustrated by the following response from a patient participant below:

“Oh, it took me about three days to go to the hospital for healthcare after noticing the lump in my breast.”  (IDI with BCP No. 13)

“Well, it took me 15 years before going to the hospital for treatment...because initially I really did not know what it was so I was managing it my own way all by myself until I realized the pain was becoming too severe and unbearable”  (IDI with BCP No. 04)

### 3.10. Experiences of Participants with BC Cure

On whether the breast cancer disease could be cured or not, the opinion expressed by the herbalists that participated in this study was that the disease is curable because they were of the view that much as there are many ailments in the world, God has made provision for the cure of all of them by making herbs available to man for this purpose, as intimated by an herbalist respondent who cited the case of a client in her 40's who saw an almost instant positive results after seeking health care at his herbal facility. This response is illustrated below.

“It is highly curable by God's grace. Although a lot of diseases abound, God has made available several herbs to cure the diseases. We herbalists have been treating several diseases with these herbs. I recall one woman in her 40's who had breast cancer came to me for assistance. Her condition was very severe and I could tell that she had spent a lot of money on orthodox medication. I applied some herbs on her breast and just 2 days after, she called to tell me that the breast had bust. She was amazed at the efficacy of the herb. So the herbs are very powerful and when rightly identified for its purpose, can cure the breast cancer disease.”  (IDI with an herbalist No. 01)

### 3.11. Participants' Experiences with Cost of BC Treatment

Participants were unanimous in their view that cost of seeking treatment for BC at the hospital is costly. They added that as a result of the high cost, they have to part with huge sums of money to purchase drugs which are supposedly subsidized from private pharmacies because the hospital pharmacy usually does not have some in stock. This is illustrated by the quote below:

“The cost of drugs for this disease  [BC] is very high. The last time I went to the hospital pharmacy they told me they did not have the drug in stock. I had to go to a private pharmacy in town to get some and this cost me 2.5 million in the old Cedi denomination  [that's about US$50.00], and I needed two of that for three months. Imagine how many of these people can afford that!?”  (IDI with BCP no. 6)

A health worker had this to say:

“Here the problem normally has to do with regular supply of drugs for managing the cancer that is for palliative care. The drugs are simply not there to give to the patients when they come. They are therefore forced by this challenge to go and buy them from outside at high prices.”  (IDI with Health worker 03)

## 4. Discussion

This study was aimed at exploring breast cancer patients' health seeking behaviour as well as their knowledge of the disease in a BC management Centre of KATH in Ghana. The findings are discussed in the context of patients' sociodemographic profile; perceptions about breast cancer burden; knowledge of causes of breast cancer; participants' knowledge of signs and symptoms of breast cancer; their perceptions about treatment and cure of breast cancer; sources of seeking healthcare for breast cancer; description of the nature and type of care given at the hospital; factors that influenced the choice of health care for breast cancer; duration between onset of BC and health seeking.

Some patients' knowledge about BC was generally low with one of them attributing the cause of her BC disease to a spider-bite that led to itching around the nipple and nipple discharge. This finding of low knowledge among BCPs is similar to that by Lehmann, Brian D., et al. 2011 [21].

### 4.1. Practice of Breast Self-Examination among Patients

The first step that triggers one into an action in seeking healthcare is the identification and recognition of signs and symptoms of the disease. For BC, the most basic and cheapest means of doing this is through breast self-examination (BSE). In this study, however, though patients generally reported knowledge of SBE, they did not practice it to enable them to seek early treatment for any unusual observations, and those who even reportedly practiced it sparingly did so. This confirms what was reported among women attending primary healthcare in Kuwait where only a few (21%) had breast self-examination [22]. There is therefore the need for vigorous and sustained community education programmes on BC to sensitize the general population especially women about the importance of breast self-examination in the preventive efforts in combating the disease.

### 4.2. Health Seeking Behaviours for BC

This study demonstrated that BC patients sought help for their condition from diverse health providers including both orthodox and unorthodox sources. This finding is consistent with a study among cancer patients in Hawaii that found patients often resort to the use of faith-based resources and complementary and alternative medicine in addition to using conventional cure from physicians [23]. These findings are also similar to that of another one conducted in Ontario, Canada, between 1998 and 2005 which revealed that the proportion of BC patients using sources other than biomedical to seek cure for BC varied from 67% to 83% [12]. This implies that the adoption of pluralistic ways of seeking health care for BC is not limited to only developing countries but is being highly practiced in the developed world where the health care system is expected to be optimal [24]. In some African communities such as Sokoto in Nigeria, cancer patients generally resorted to faith-based and traditional medical practice as their primary sources of healthcare for BC other than the orthodox means [25–27] This health seeking behaviour may pose delays and risk in the management of BC [28, 29] especially where the various providers do not have the skills to manage BC. The delays are likely to be worse if the first point of care lacks the knowledge to diagnose or suspect BC [30].

The role of husbands as the decision-makers in determining where and when to seek health care for BC cannot be overemphasised as most of the patients who participated in this study mentioned their husbands as the final decision-makers when it came to seeking healthcare for their BC condition. The response below illustrates this.

“…it was my husband's to go and seek health care from the hospital. I did not challenge him on that because he was going to pay the bill.”  (IDI with BCP No. 16)

The patients who participated in this study were mostly peasant farmers and petty traders whose earnings may be little compared to the cost of BC treatment. They therefore rely on other sources such as funds from their partners. It was also evident that the health care decision-making of BC patients was limited to that of their partners who support their health care financial needs. This finding strongly affirms those reported by other studies that women with low socioeconomic status tend to largely rely on their husbands for financial access to their healthcare [31]. This observation may lead to delays in seeking healthcare and further complications as found by Pinder, Nzayisenga et al. 2018 [32]. Indeed, a study conducted in Kumasi, Ghana, between 2004 and 2009 showed that 60% and 85.2% of BC patients presented late with Stages III and IV, respectively of BC [19]. The reasons cited for this behaviour include low socioeconomic status which limits a woman's social and financial freedom [33, 34]. Health promotion strategies aimed at getting BC patients at seeking early and appropriate healthcare should consider this gate-keeping position of men and bring them on board to achieve prompt patient care.

The low economic status of the BC patients reported by this study compared to the high cost of treatment for BC brings to the fore the need for governments in developing countries to develop effective interventions that would prioritise BC treatment to expand access [35, 36]. Whereas as per the national policy the diagnosis and treatment of BC in Ghana are supposed to be covered fully under the national health insurance scheme (NHIS), reports by all the participants in this study indicated that the scheme does not cater for the full cost of treatment. Participants were unanimous that most of the times there are no drugs available at the dispensary for patients thereby forcing patients to go to private pharmacies to buy them at very exorbitant prices from private shops. These situations offer no choice to patients who resort to other means of treatment considered to be less expensive compared to the orthodox mode of treatment [37].

Patients' perceptions of chronic diseases such as BC diagnosis and management influence healthcare seeking behaviours [29]. In this study some patients perceived the cause of BC to be of spiritual origin. In such instances, BC patients are likely to resort to spiritual means to get cured [23]. We strongly recommend an urgent need for the development and adoption of an integrative approach to BC management by health authorities. This approach should include alternative medicine practitioners such as herbalists and faith healers who expressed their preparedness to collaborate with those in the orthodox. This approach will make it possible to win the confidence of these practitioners and get them to easily refer clients who visit them with suspected BC to the hospital for clinical screening and diagnosis to be done thereby reducing the incidence of late reporting and diagnosis; because whether we like it or not their services will be patronised anyway.

## 5. Limitation

The BC patients may be seeking to suggest that the use of the hospital was their first point of call for health care for their condition because perhaps they were not sure what the consequences of opening up and admitting to resorting to the use of herbalists as first points of call might possibly turn out to be, especially considering the fact that this study was facility-based. It would therefore be better for future studies to focus on community-based rather than facility-based participants since the former is less intimidating for participants to speak up and express concerns freely [38].

## 6. Conclusion

Breast cancer patients (BCPs) in this study resorted to both orthodox and unorthodox sources for treatment. Though the hospital was generally the first point of care, patients reported late with advanced stages of BC for treatment due mainly to misinformation about the disease. The high cost of BC treatment at the hospital deters BCPs from sticking solely to the orthodox system through their BC management journey. Men (particularly husbands) were the main decision-makers regarding when and where to seek treatment for their partners BC condition. Public education on early detection of BC, health providers' engagement on appropriate detection, and referral and health care financing on BC is highly recommended.

## Figures and Tables

**Table 1:** Breakdown of interviews conducted.

Category of participants	Interview type	Number of interviews
Breast Cancer patients	In-depth interview	20

Health workers	In-depth interview	5

Caregivers	In-depth interview	8

Herbalists	In-depth interview	2

*Total *		*35*

**Table 2:** Participants' social and demographic profile (N = 35).

Demographic Characteristics	BC Patients (N=20) n (%)	Care-givers (N=8) n (%)	Health Workers (N=5) n (%)	Herbalists/Traditional Healers (N=2) n (%)
Age				

18 – 30	1 (5)	3 (37.5)	1 (20)	0 (0.0)

31 – 60	17 (85)	5 (62.5)	4 (80)	2 (100)

60+	2 (10)	0 (0.0)	0 (0.0)	0 (0.0)

Occupation				

Unemployed	0 (0.0)	0 (0.0)	0 (0.0)	0 (0.0)

Farming	5 (25)	3 (37.5)	0 (0.0)	0 (0.0)

Teaching	3 (15)	0 (0.0)	0 (0.0)	0 (0.0)

Trading	11 (55)	2 (25)	0 (0.0)	0 (0.0)

Nursing	1 (5)	0 (0.0)	2 (40)	0 (0.0)

Medical physicist	0 (0.0)	0 (0.0)	3 (60)	0 (0.0)

Student	0 (0.0)	2 (25)	0 (0.0)	0 (0.0)

Other	0 (0.0)	1 (12.5)	0 (0.0)	2 (100)

Educational Level				

No formal education	4 (20)	0 (0.0)	0 (0.0)	0 (0.0)

Basic	12 (60)	5 (62.5)	0 (0.0)	2 (100)

Secondary	1 (5)	0 (0.0)	0 (0.0)	0 (0.0)

Tertiary	3 (15)	3 (37.5)	5 (100)	0 (0.0)

Marital Status				

Single	0 (0.0)	3 (37.5)	3 (60)	0 (0.0)

Married	18 (90)	5 (62.5)	2 (40)	2 (100)

Widowed	2 (10)	0 (0.0)	0 (0.0)	0 (0.0)

Religion				

Christianity	19 (95)	7 (87.5)	5 (100)	2 (100)

Islam	1 (5)	1 (12.5)	0 (0.0)	0 (0.0)

Other	0 (0.0)	0 (0.0)	0 (0.0)	0 (0.0)

Sex				

Female	20 (100)	6 (75)	1 (20)	0 (0.0)

Male	0 (0.0)	2 (25)	4 (80)	2 (100)

## Data Availability

The qualitative data used to support the findings of this study are available from the corresponding author upon request.
